# Does the Mediterranean Diet Play a Beneficial Role in Managing the Health of Overweight/Obese Breast Cancer Survivors?

**DOI:** 10.3390/nu16234214

**Published:** 2024-12-06

**Authors:** Syeda Maria Yaqoob, Layla Haidar, Marlyn A. Allicock, Natalia I. Heredia

**Affiliations:** Department of Health Promotion & Behavioral Sciences, School of Public Health, University of Texas Health Science Center at Houston, Houston, TX 77030, USA; syeda.m.yaqoob@gmail.com (S.M.Y.); layla.haidar@uth.tmc.edu (L.H.); marlyn.a.allicock@uth.tmc.edu (M.A.A.)

**Keywords:** cancer, obesity, Mediterranean diet

## Abstract

**Background:** Numerous studies have established a correlation between the Mediterranean diet and a reduced risk of breast cancer, as well as its efficacy in supporting weight management. Notably, obesity is widely recognized as a significant risk factor for the development of breast cancer. The Mediterranean diet has been shown to improve health outcomes among overweight or obese breast cancer survivors. This narrative review aims to consolidate information on the existing research interventions elucidating the benefits of the Mediterranean diet for the overall health of breast cancer survivors. **Methods:** Using the search terms “Mediterranean diet” and “breast cancer survivor”, a total of 44 articles were identified. This review focuses on the six articles meeting the inclusion criteria, examining impacts on various health outcomes such as weight loss, quality of life, and various metabolic parameters (e.g., triglycerides, BMI, fasting glucose). **Results:** Most of the intervention studies examined weight loss and metabolic parameters including BMI and fat mass. The research results indicate that the Mediterranean diet effectively reduces BMI, waist circumference, and fat mass. Moreover, the data suggest that this dietary approach may support attaining a healthier BMI in overweight or obese breast cancer survivors. The data from various studies show no statistically significant findings for high-density lipoprotein, low-density lipoprotein, and total cholesterol levels. The assessment of quality of life varied across the studies, leading to challenges in reaching definitive conclusions. **Conclusions:** This narrative review offers a comprehensive overview of the impact of the Mediterranean diet on the health outcomes of breast cancer survivors who are overweight or obese.

## 1. Introduction

Cancer remains one of the leading causes of death worldwide [1]. Among females, breast cancer is the second most common cause of cancer-related mortality [2]. It is estimated that approximately 310,720 new cases of invasive breast cancer will be diagnosed in 2024 [2]. Several risk factors contribute to breast cancer, including age, genetics, physical inactivity, excessive alcohol consumption, smoking, inadequate vitamin supplementation, processed food consumption, chemical exposure, and being overweight or obese [3]. Obesity is recognized as a significant risk factor for developing breast cancer, particularly among postmenopausal women; in these women, obesity can increase the risk of developing breast cancer by approximately 30% to 60% compared to women of normal weight [4,5]. Notably, higher Body Mass Index (BMI) has been associated with more aggressive tumor characteristics such as increased lymph node metastasis and larger tumor size [3]. Individuals who are overweight or obese with a history of breast cancer not only face an increased risk of therapy-related complications and disease recurrence but also experience negative impacts on quality of life, including issues such as neuropathy, negative body image, cardiotoxicity, sexual dysfunction, chronic fatigue, and lymphedema [6].

Diet plays a significant role in reducing toxicity, enhancing chemotherapy’s efficacy, strengthening the immune system, promoting weight management, and mitigating long-term complications in cancer patients [7]. With the understanding that diet holds immense potential in improving outcomes for cancer patients, it is critical to explore specific dietary patterns that have shown promising results. One diet that has gained significant attention in the context of breast cancer is the Mediterranean diet [8]. The Mediterranean diet has been associated with a reduced risk of breast cancer and has been successful in helping patients lose and maintain a healthy weight [8]. The Mediterranean diet comprises a high intake of fresh fruits, vegetables, legumes, and whole grains [9] and moderate consumption of fish, poultry, dairy products, and red wine [9]. The Mediterranean diet emphasizes the intake of mono-unsaturated fatty acids and the restriction of saturated fatty acids, typically found in red meat [8]. Yogurt and nuts are additional important components of this diet, and in general, the diet is rich in antioxidants and nutrients with anti-inflammatory properties [7]. Importantly, the Mediterranean diet is not overly restrictive but is rather a broader philosophy that emphasizes balanced meals, social dining, and mindful eating practices, guiding one’s approach to food and its utilization [10].

There is a strong correlation between adherence to the Mediterranean diet and a decreased risk of breast cancer and reduced mortality rates [11]. A prospective cohort study conducted in northern Italy demonstrated that women who adhered more closely to the Mediterranean diet had a significantly better breast cancer prognosis; the overall survival rate was 63.1% for those with high adherence compared to 53.6% for those with low adherence [11]. Similarly, findings from a Netherland Cohort Study on postmenopausal breast cancer showed an inverse association between adherence to the Mediterranean diet and receptor-negative breast cancer [12]. These findings collectively suggest the potential benefits of the Mediterranean diet in reducing the risk of breast cancer and improving outcomes for breast cancer patients. However, how the diet is related to breast cancer outcomes in women with an elevated BMI has not been well explored. The aim of this narrative review is to summarize the existing research interventions examining the impacts of the Mediterranean diet on the overall health of breast cancer survivors who are overweight or obese, specifically focusing on its effects on weight loss, metabolic parameters, and quality of life.

## 2. Methods and Materials

### 2.1. Literature Search

The articles found for this narrative review were identified from March to August 2023 using PubMed. PubMed was chosen due to its extensive indexing of health and medical research relevant to the Mediterranean diet, breast cancer survivors, and obesity-related health outcomes. The key search terms included “Mediterranean diet” and “breast cancer survivor”.

### 2.2. Study Selection

Studies were considered eligible if (1). the study participants were breast cancer survivors; (2). the study participants were overweight or obese; (3). the study examined a Mediterranean diet intervention; and (4). the study included at least one of the following outcomes: weight loss, metabolic parameters, or quality of life. Exclusion criteria were used to remove studies that did not meet these parameters. The preliminary screening of studies was completed by reviewing the titles and abstracts to assess their eligibility. The full-text articles were reviewed for the remaining studies to determine their inclusion. It is worth noting that while some articles included other diet types in the study (e.g., Mediterranean vs. low-fat diet), this narrative review exclusively focuses on the Mediterranean diet groups in each study.

**Figure 1:**
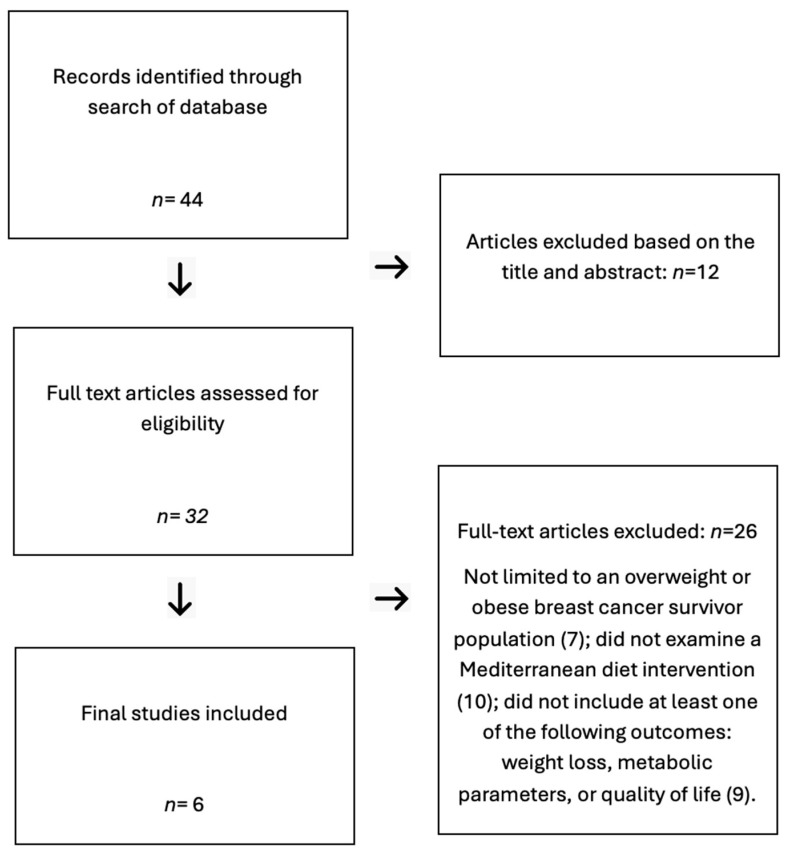
A diagram showing the selection process for articles.

Following the literature search, 44 articles were identified. After 38 articles were excluded, 6 articles met the inclusion criteria. Figure 1 outlines the selection process used.

## 3. Results

### 3.1. Description of Each Study (Table 1)

Cho et al. (2020) [13]

This was an 8-week pilot randomized control trial (RCT) conducted in South Korea that investigated the effects of pairing the Mediterranean diet with naltrexone/bupropion treatment for weight loss in breast cancer survivors with a BMI greater than 25.0 kg/m^2^. Forty-four overweight or obese breast cancer survivors were divided into two groups; one group received the Mediterranean diet alone (the group whose data were examined for this review; *n* = 21) and the other received the Mediterranean diet along with naltrexone/bupropion medication. Additionally, a third group of 28 non-cancer, age-matched participants received the Mediterranean diet combined with naltrexone/bupropion medication. The study examined changes in body weight, nutrient intake, quality of life, and metabolic parameters in participants from all three groups.

Parma et al. (2022) [10]

This was a 1-year RCT in the U.S. examining the effect of an anti-inflammatory Mediterranean dietary intervention on the quality of life of breast cancer survivors. A sample of 153 adult (18 and older) breast cancer survivors with elevated BMI (≥25.0 kg/m^2^) was randomly assigned to control and intervention groups. The intervention group received six monthly workshops that included culinary demonstrations, meal planning, recipes, and 12 motivational interviewing telephone calls designed to promote the adoption and maintenance of healthier dietary habits based on the Mediterranean diet. The control group received monthly informational brochures from the American Institute for Cancer Research and non-motivational interviewing telephone calls prior to their assessment appointments. The study examined quality of life indicators through the Perceived Stress Scale (PSS), the Functional Assessment of Cancer Therapy—General (FACT-G) and Breast Cancer (FACT-B), and the Center for Epidemiologic Studies Depression Scale (CES-D).

Pellegrini et al. (2020) [14]

This was a 16-week randomized pilot intervention trial conducted in Italy that compared the effectiveness of a Mediterranean diet and probiotics (intervention) versus a Mediterranean diet alone (control) in altering the gut microbiota and metabolic profile in obese breast cancer survivors. The study was open label; thus, the participants knew their group assignment. The sample of 34 breast cancer survivors with a BMI between 25.0 kg/m^2^ and 35.0 kg/m^2^ was randomly assigned to control (*n* = 18; group that were examined for this review) and intervention groups. The study included anthropometric and nutritional assessments and assessed adherence to the Mediterranean diet, compliance with physical activity, and metabolic parameters.

Finocchiaro et al. (2016) [15]

This was a single-arm intervention study in Italy that tested the effect of a 1-month educational program focused on promoting dietary change in alignment with the Mediterranean diet on weight loss in breast cancer survivors. A sample of 100 breast cancer survivors with elevated BMI (≥25.0 kg/m^2^) was recruited for the study. The participants attended four classes over one month, which included training sessions, lectures, and workshops. The study examined anthropometric data (BMI, waist circumference) and behaviors, including physical activity, dietary habits, and food intake patterns at 2- and 6-month follow-ups.

Braakhuis et al. (2017) [16]

This was a 6-month RCT conducted in New Zealand analyzing the effects of dietary nutrition education on weight and health biomarkers in stage 1–3 breast cancer survivors. The sample was 50 adult women with elevated BMI >25 kg/m^2^). This study tested two interventions; the first intervention group focused on a low-fat diet, while the second intervention group emphasized the Mediterranean diet (the group whose data were examined for this review; *n* = 17), and both groups received six-session education packages to support dietary changes. The control group did not receive any intervention. This study examined BMI, waist circumference, blood biomarkers, and quality of life.

Gnagnarella et al. 2023 [17]

This was an RCT conducted in Milan, Italy, to evaluate the effectiveness of a 6-month weight loss intervention. The sample consisted of 231 adult breast cancer survivors with elevated BMI (≥25 kg/m^2^). The participants were randomized into four groups: dietary intervention based on the Mediterranean dietary pattern (the group whose data were examined for this review; *n* = 56), physical activity intervention, combined physical activity and dietary intervention, and a comparison group receiving only general information and recommendations for a healthy lifestyle.

### 3.2. Populations

Apart from all being overweight or obese breast cancer survivors, the populations studied were diverse, encompassing various age groups, geographical locations, and health statuses, providing a comprehensive overview of the Mediterranean diet’s impact across different demographics. The research conducted by Cho et al. [13]. occurred at a hospital in Seoul, South Korea. Three studies focused on Italian participants, with Gnagnarella et al. [17] recruiting from Milan, Italy; Pelligrini et al. [14] from the Breast Unit in Turin, Italy; and Finocchiaro et al. [15] from Turin, Italy. The participants for the latter study were enlisted by the Department of Clinical Nutrition through hospital staff responsible for the follow-up care of breast cancer patients. According to Parma et al. [10], their study included 43% White and 41% Latino individuals from San Antonio, Texas, USA. Braakhuis et al. [16] conducted their study with post-menopausal women from Auckland, New Zealand, who had been at least three months removed from active treatment. While all studies were with adults (ages 18 and older), the age range varied between the studies; Cho et al. [13] limited their sample to 20–65 years and Braakhuis et al. [16] limited it to 50 years and over, whereas Pelligrini et al. [14] and Finocchiaro et al. [15] had a broader age range of 18–70 years and Gnagnarella et al. [17] broadly included women that were 18 years or older.

### 3.3. Study Designs

The existing studies focused on the Mediterranean diet in overweight and obese breast cancer survivors include four RCTs: two 6-month-long RCTs [16,17], an 8-week RCT [13], and a 1-year-long RCT [10]. Additionally, there was a 1-month single-arm intervention with follow-up at 2 and 6 months [15] and a 16-week randomized pilot intervention trial [14].

#### 3.3.1. Measurement of Outcomes

The studies focused on several health outcomes, including weight loss measured in kilograms [13,14,15,16,17], quality of life factors assessed using self-reported questionnaires such as the Perceived Stress Scale (PSS), the Functional Assessment of Cancer Therapy—General (FACT-G) and Breast Cancer (FACT-B), the Center for Epidemiologic Studies Depression Scale (CES-D), the European Organization for Research and Treatment of Cancer quality of life questionnaire (EORTC QLQ-C30), the obesity-related problems scale, and the Functional Assessment of Chronic Illness Therapy—Fatigue (FACIT-F) subscale. Additionally, anthropometric variables were used as outcomes, including the BMI in kilograms per square meter (kg/m^2^), as well as metabolic parameters, including the fat mass in kilograms, fasting glucose in milligrams per deciliter (mg/dL), insulin levels, homeostasis model assessment of insulin resistance (HOMA-IR), triglyceride levels in mg/dL, fat percentage, total cholesterol, high-density lipoprotein (HDL) in mg/dL, and low-density lipoprotein (LDL) in mg/dL (Table 2). We describe each in detail below.

#### 3.3.2. Weight and BMI

Among the six studies reviewed, weight loss was a primary focus in five [13,14,15,16,17]. In all five studies, a decrease in weight was observed for Mediterranean diet group participants, with four studies demonstrating a statistically significant decrease. Specifically, Gnagnarella et al. [17] reported a significant weight loss for Mediterranean diet group participants of an average of 4.7 kg at six months as compared to a control group that lost 1.7 kg (3 kg difference). Finocchiaro et al. [15] reported a significant weight loss of an average of 3.4 kg at six months (*p* < 0.001), followed by Pellegrini et al. [14], with a weight loss of an average of 3.1 kg at four months (*p* < 0.015), and Cho et al. [13], with a weight loss of 1.8 kg on average after two months (*p* < 0.05), though the changes in these three studies describe pre- to post-test comparison within the Mediterranean diet groups. Braakhuis et al. [16] reported an average decrease of 1.61 kg at six months, although this change was not statistically significant.

Out of the six studies examined, four investigated BMI. Cho et al., Pellegrini et al., and Finocchiaro et al. [13,14,15] observed a significant decrease in BMI (0.7 kg/m^2^, 1.3 kg/m^2^, and 2.0 kg/m^2^, respectively) when looking at pre- to post-test BMI in the Mediterranean diet groups only. Furthermore, Braakhuis et al. [16] showed a mean decrease in BMI of 1.02 kg/m^2^ and showed this to be statistically significant when compared to a control group.

#### 3.3.3. Metabolic Parameters

Five out of the six studies described in this review assessed metabolic parameters as outcomes. In Braakhuis et al.’s [16] study, there was a non-statistically significant decrease in HbA1c, total cholesterol, and LDL in the Mediterranean diet group, as well as a statistically significant decrease in waist circumference and BMI relative to the control. Cho et al. [13] reported statistically significant reductions in fat mass, fasting glucose, insulin, HOMA-IR, and triglycerides (*p* < 0.05) for the Mediterranean diet group but no statistically significant differences in waist circumference, total cholesterol, HDL, or LDL. Pellegrini et al. [14] found a significant decrease in fasting glucose and HOMA-IR (*p* < 0.05) in the Mediterranean diet group, but no statistically significant changes in insulin, triglycerides, waist circumference, total cholesterol, HDL, or LDL. Gnagnarella et al. [17] examined glucose levels, fat mass, total cholesterol, HDL, LDL, triglycerides, waist circumference, insulin, and HOMA-IR, finding a statistically significant decrease only in waist circumference and fat mass for the Mediterranean diet group as compared to a control group. In the study conducted by Finocchiaro et al. [15], there was a statistically significant decrease (*p* < 0.001) in waist circumference for the Mediterranean diet group.

#### 3.3.4. Quality of Life

Among the six studies included in this review, three investigated the impact on quality of life [10,13,16]. The measures to assess quality of life varied across the studies, and the findings were inconsistent. Cho et al. [13] observed a statistically significant improvement (*p* < 0.05) in quality of life after a 2-month Mediterranean diet-only intervention, as assessed by self-reported European Organization for Research and Treatment of Cancer quality of life questionnaire (EORTC QLQ-C30) global health status, physical functioning, cognitive functioning, and social functioning subscales, but not as assessed by the other EORTC QLQ-C30 subscales (role functioning and emotional functioning), the obesity-related problems scale and the Functional Assessment of Chronic Illness Therapy—Fatigue (FACIT-F) subscale. Parma et al. [10] conducted a 1-year culinary-based intervention focused on promoting the Mediterranean diet and assessed quality of life using the Perceived Stress Scale (PSS), the Functional Assessment of Cancer Therapy—General (FACT-G) and Breast Cancer (FACT-B), and the Center for Epidemiologic Studies Depression Scale (CES-D). Their analysis revealed a reduction in perceived stress within the intervention group at the 6-month follow-up; however, this effect had dissipated by the 12-month follow-up. None of the other quality of life indicators, such as FACT-G and FACT-B, yielded significant results. In the study conducted by Braakhuis et al. [16], a 6-month Mediterranean diet intervention resulted in no significant difference in FACT-B quality of life questionnaire scores between the pre- and post-intervention measurements in the Mediterranean diet group.

**Table 1:** Description of studies.

Reference	Intervention	Design	Setting	Population
Cho et al. 2020 [13]	- Received dietary training from professional nutritionists at the baseline visit and samples of a Mediterranean diet three times during the trial	8-week pilot randomized control trial	Recruited from Gangnam Severance Hospital in Seoul, South Korea, from July 2017 to July 2018	- Breast cancer survivors - 20 women aged 20–65 years with a BMI of >25.0 kg/m^2^
Pellegrini et al. 2020 [14]	- A personalized MD developed by trained dietician	16-week pilot intervention trial	Recruited from the Breast Unit at San Lazzaro Hospital in Turin, Italy, from January 2017 to January 2018	- Breast cancer survivors - 18 women aged 18–70 years with a BMI between 25.0 kg/m^2^ and 35.0 kg/m^2^
Finocchiaro et al. 2016 [15]	- Consisted of four meetings once a week for 2 h including nutrition (focused on Mediterranean diet) and physical activity lectures, training sessions, and workshops	1-month single arm intervention	Recruited from the Department of Clinical Nutrition Hospital in Turin, Italy, from September 2009 to March 2010	- Breast cancer survivors - 100 women aged 18–70 years with a BMI of ≥25.0 kg/m^2^
Parma et al. 2022 [10]	- The intervention group attended 6 monthly workshops (lectures on anti-inflammatory topics and chef-prepared food demonstrations) and received monthly newsletters and telephone calls incorporating motivational interviewing - The control group received monthly nutritional brochures from the American Institute for Cancer Research.	1-year randomized control trial	Recruited from the Cancer Therapy and Research Center in San Antonio, Texas	- Breast cancer survivors - 153 women aged >18 years with a BMI of >25.0 kg/m^2^
Braakhuis et al. 2017 [16]	- Dietary Arm 1 was the MD diet; they received six group nutrition and lifestyle education sessions from the same educator once a month with a summary newsletter sent two weeks thereafter.	6-month randomized control trial	Recruited from Aukland, New Zealand	- Breast cancer survivors - 15 women aged >50 years with a BMI of >25.0 kg/m^2^
Gnagnarella et al. 2023 [17]	- The dietary intervention group received personalized counselling focused on the MD diet and, motivational meetings with a psychologist focused on goal setting and psychological support.	6-month randomized control trial	Recruited from Milan, Italy	- Breast cancer survivors - 56 women aged >18 with a BMI of >25.0 kg/m^2^

**Table 2:** Health outcomes for each study.

Reference	Weight Loss (kg)	Quality of Life Factors	Metabolic Factors									
			BMI (kg/m^2^)	Fat Mass (kg)	Fasting Glucose (mg/dL)	Insulin	HOMA-IR	Triglyceride (mg/dL)	Waist Circumference (cm)	Total Cholesterol	HDL (mg/dL)	LDL (mg/dL)
Cho et al. 2020 [13]	−1.8 *	Improved *	−0.7 *	−1.8 *	−7.0 *	1.10 *	−0.4 *	−25.0 *	No sig	No sig	No sig	No sig
Pellegrini et al. 2020 [14]	−3.1 *	N/A	−1.3 *	N/A	−6.8 *	No sig	−0.8 *	No sig	No sig	No sig	No sig	No sig
Finocchiaro et al. 2016 [15]	−3.4 *	N/A	−2.0 *	N/A	N/A	N/A	N/A	N/A	−5.3 *	N/A	N/A	N/A
** Parma et al. 2022 [10]	N/A	Did not Improve	N/A	N/A	N/A	N/A	N/A	N/A	N/A	N/A	N/A	N/A
** Braakhuis et al. 2017 [16]	No sig	Did not improve	−1.02 *	N/A	N/A	N/A	N/A	No sig	−1.40 *	No sig	No sig	No sig
** Gnagnarella et al. 2023 [17]	−4.7 *	N/A	N/A	−11.6 *	No sig	No sig	No sig	No sig	−4.3 *	No sig	No sig	No sig

* = statistical significance; “−” = decreased number; N/A = did not test in the study; “No sig” = was not statistically significant in the study, ** = statistical significance as compared to a control.

## 4. Discussion

The primary objective of this narrative review was to summarize existing research interventions on the benefits of a Mediterranean diet in overweight or obese breast cancer survivors, with a specific focus on weight loss, metabolic parameters, and quality of life. The findings of this narrative review demonstrate that the Mediterranean diet has a significant impact on weight loss, BMI, and fat mass in breast cancer survivors who are overweight or obese. This is consistent with findings in other populations with obesity like older adults, post-menopausal women, and individuals who have not suffered from breast cancer [4,5,18,19,20].

The effects of the Mediterranean diet on fasting glucose, insulin, HOMA-IR, triglyceride levels, and waist circumference varied across the studies in this review, with some changes reaching statistical significance and others not, making it hard to draw definitive conclusions. Notably, three metabolic parameters (HDL, LDL, and total cholesterol) consistently showed no significance in any of the studies, suggesting that the Mediterranean diet may not have a significant impact on these particular metabolic variables or at least not in the timeframe studied. This finding is inconsistent with the literature. The results of a systematic review of 58 studies concluded that triglyceride levels were lower and HDL levels were greater in participants who adhered to the Mediterranean diet [21,22,23,24]. Additionally, a study of cholesterol homeostasis in men with metabolic syndrome showed that the Mediterranean diet reduced LDL concentrations [25]. It is possible that the null findings for these three metabolic parameters in intervention studies to date could be due to the effects of chemotherapy or other medications taken during breast cancer treatment [26,27], or the need for longer study periods to see effects. However, additional research will be needed.

Regarding quality of life, the measures used across the studies were different and the findings were inconsistent, making it difficult to draw conclusions. However, based on the studies examined, it appears that the Mediterranean diet does not significantly improve quality of life in breast cancer survivors. This is not consistent with what the literature shows for other populations. A significant link has been observed between adherence to the Mediterranean diet and quality of life for adults [18,28,29,30,31]. The Mediterranean diet has also been associated with a reduced risk of depression and anxiety for adults [32,33,34].

Research has demonstrated that adherence to the Mediterranean diet is associated with various favorable metabolic parameters, even outside the context of breast cancer. Bertoli et al. demonstrated that among Non-Hispanic White subjects, adherence to the Mediterranean diet was linked to reductions of −0.118 kg/m^2^ in BMI, −0.292 cm in waist circumference, −0.002 cm/cm in waist-to-height ratio, −1.125 mm in the sum of four skinfold measurements, and −0.045 cm in visceral abdominal tissue [35]. Furthermore, adherence to the Mediterranean diet served as a protective factor against obesity and excess visceral adipose tissue [35]. Greco et al. demonstrated that adherence to the Mediterranean diet in obese individuals led to a significant decrease in body weight and BMI (both *p* < 0.0001), as well as reductions in insulin levels (*p* = 0.037), HOMA-IR (*p* = 0.026), and leptin levels (*p* = 0.008) [36]. The Mediterranean diet has also been associated with significant improvements in various health metrics among patients with type 2 diabetes [37]. In a study by Huo et al., consumption of the Mediterranean diet resulted in a decrease in HbA1c levels by 0.30%, fasting plasma glucose by 0.72 mmol/L, fasting insulin levels by 0.55 μU/mL, total cholesterol by 0.14 mmol/L, triglycerides by 0.29 mmol/L, systolic blood pressure by 1.45 mm Hg, and diastolic blood pressure by 1.41 mm Hg [37]. Kastorini et al. reported a reduction of 0.42 cm in waist circumference, a decline in triglyceride levels by 6.14 mg/dL, and an increase of 1.17 mg/dL in HDL levels [38]. Additionally, blood pressure measurements decreased, with systolic pressure dropping by 2.35 mm Hg and diastolic pressure falling by 1.58 mm Hg [38].

### 4.1. Directions for Future Research

To enhance the robustness of evidence synthesis, we suggest the use of standardized outcome measures, a more homogeneous target population, and more robust interventions like randomized control trials that compare the Mediterranean diet to a control group would be helpful in drawing definitive conclusions and improve comparability. Most of the aforementioned research studies were conducted on individuals from specific ethnic backgrounds, potentially causing an overemphasis on one ethnic group in the study results. Therefore, it is critical to conduct similar research studies within the U.S. to determine whether the findings are generalizable to the more racially/ethnically diverse overweight and obese breast cancer survivors in the U.S. population. Furthermore, the studies had varying durations, which could impact the reliability of the results. It is essential to ensure consistency in the duration of future studies because consistent study durations allow for more reliable comparisons and conclusions across different research findings. According to Golan et al. (2012) [39], changes in metabolic and other biometric parameters like C-reactive protein start appearing within 6 months of dietary changes and continue to happen over the course of 2 years. Additionally, it is important to examine the distinctions between pre-menopausal and post-menopausal women to achieve a more comprehensive understanding of this specific population.

### 4.2. Strengths and Limitations

Several limitations should be considered regarding this study. Firstly, the studies that were examined had different lengths of intervention. The six studies included in this narrative review ranged from 1 month to 1 year in duration, which could have influenced the results. According to the National Cancer Institute, the definition of “breast cancer survivor” is a patient who has ever been diagnosed with breast cancer, and they are a cancer survivor from the time of diagnosis through the remainder of their life. However, since this review included studies from outside the U.S., this definition may vary slightly across articles, potentially impacting the results due to variations in populations. The inclusion/exclusion criteria for participants for each study in the review were also different (e.g., the duration of being cancer-free). Another limitation of this review is the variability in interventions among the six studies, which can affect the study design, quality, potential for bias and confounding, and comparability of results. For instance, some studies solely provided dietary guidelines for participants to follow, while others included educational workshops and hands-on nutrition and cooking sessions. However, the study populations remained cohesive by including adult women with a BMI greater than or equal to 25.0 kg/m^2^. Furthermore, although the measurement of health outcomes differed across the studies, most outcomes were assessed similarly using blood tests for metabolic parameters and approved protocols for measuring body weight, alongside validated, self-reported measures for quality of life. Despite these limitations, this narrative review offers an overview of the existing literature on the impact of the Mediterranean diet on health outcomes in breast cancer survivors who are overweight or obese.

## 5. Conclusions

This narrative review examined the effects of the Mediterranean diet on breast cancer survivors who are overweight or obese. We found that in most studies, there was statistically significant weight loss, as well as reductions in BMI, fat mass, and waist circumference. However, further research is necessary to investigate the long-term effects of adhering to the Mediterranean diet in this population and its influence on other metabolic and quality of life factors.

## Data Availability

No new data were collected.
